# The Fate of Borderline Pathology in Dimensional Classification Systems: A Narrative Review

**DOI:** 10.3390/brainsci16030326

**Published:** 2026-03-19

**Authors:** Danilo Pesic, Dusica Lecic-Tosevski, Bojana Pejuskovic, Ana Munjiza-Jovanovic, Olivera Vukovic

**Affiliations:** 1 Institute of Mental Health, 11000 Belgrade, Serbia; danilo.pesic@imh.org.rs (D.P.); bojana.pejuskovic@imh.org.rs (B.P.); ana.munjiza@imh.org.rs (A.M.-J.); olivera.vukovic@imh.org.rs (O.V.); 2 Faculty of Medicine, University of Belgrade, 11000 Belgrade, Serbia; 3 Serbian Academy of Sciences and Arts, 11000 Belgrade, Serbia

**Keywords:** borderline pathology, borderline personality organization, borderline personality disorder, borderline pattern descriptor, level of personality functioning, ICD-11 classification, severity, general factor of personality disorder

## Abstract

**Highlights:**

**What are the main findings?**

• Borderline symptoms do not form a distinct factor in structural analyses but consistently load on a general personality disorder factor, functioning as markers of overall severity of personality dysfunction rather than defining a separate diagnostic entity.
• The concept of the borderline level of personality functioning converges with the Level of Personality Functioning (Criterion A) in DSM-5 AMPD and the severity continuum in ICD-11, demonstrating that borderline pathology represents a dimension of structural vulnerability across personality disorders.

**What are the implications of the main findings?**

• Retaining borderline pathology as a recognizable clinical pattern within dimensional classification systems preserves continuity with established treatment and research traditions while providing clinicians with a meaningful marker of moderate-to-severe personality dysfunction.
• Dimensional assessment of personality disorders should integrate both severity rating and trait-based description, with borderline features serving as clinically useful indicators of regression proneness and the need for specialized psychotherapeutic intervention.

**Abstract:**

Recent revisions of personality disorder (PD) classifications have moved from categorical diagnoses toward dimensional models, raising renewed questions about the nosological status and clinical utility of borderline personality disorder (BPD). This narrative review traces the development of the borderline construct from early descriptions of patients positioned between neurosis and psychosis, through its theoretical consolidation within the concept of borderline personality organization, to the operationalization of BPD in DSM-III and subsequent diagnostic revisions. A central section summarizes contemporary controversies regarding the validity and utility of BPD features. Arguments for abandoning the diagnosis emphasize the absence of a distinct borderline factor in factor analytic studies, the tendency of the construct to capture fluctuating symptoms and patterns of behaviour rather than stable maladaptive personality traits, the stigmatizing and non-selective use of the label, and the lack of disorder-specific treatment approaches. In contrast, converging evidence supports the view that core borderline symptoms frequently function as markers of general PD pathology and of the severity of impairments in self and interpersonal functioning. The paper integrates the concept of the borderline level of personality functioning, conceptualizing borderline pathology as a dynamic dimension of dysfunction with potential transient regressions, and links this concept to the Level of Personality Functioning (LPF, Criterion A) within the DSM 5 Alternative Model for Personality Disorders (AMPD). Retaining borderline pathology as a dimension may support contemporary PD assessment by offering a clinically recognizable marker of overall dysfunction, a guide for rating severity, an indicator of personality structure and need for psychotherapy, without disrupting continuity with an extensive clinical and research tradition.

## 1. Introduction: Nosological History

Dimensional reforms of personality disorders (PD) classifications have challenged the status of long-standing categorical diagnoses, with BPD becoming a focal point of debate. Within official diagnostic classifications, the term borderline first appeared in the diagnosis of BPD, introduced as a distinct entity in DSM-III (1980), largely through the work of Spitzer and colleagues, building on earlier research by Gunderson and Kernberg’s concept of borderline personality organization (BPO) [1]. Spitzer defined criteria for separating a class of severely disturbed patients into two groups, borderline and schizotypal personality disorder, and early findings suggested that BPD could be differentiated from schizophrenia, depression, and other PDs by specific identity disturbances and interpersonal problems [2]. In ICD systems, the construct appeared in ICD-10 within emotionally unstable PD, with borderline and impulsive subtypes [3]. In DSM-5, the categorical model was retained, while the AMPD introduced a hybrid approach that includes a limited set of six PD categories: Antisocial, Avoidant, Borderline, Narcissistic, Obsessive-Compulsive, and Schizotypal PD, combined with a dimensional assessment of personality functioning and pathological traits. In ICD-11, categorical PD diagnoses were removed and replaced by a severity-based model with trait domain qualifiers, Negative Affectivity, Detachment, Dissociality, Disinhibition, and Anankastia, alongside the optional Borderline Pattern qualifier, which was included to preserve continuity with prior clinical and research traditions [4].

Looking at the period preceding formal classification systems, the term borderline first appeared in 1938, in Stern’s description of the “borderline group of neurosis, referring to patients who could not be clearly assigned to either the schizophrenic or the psychoneurotic group [5]. Subsequent labels such as pseudoneurotic schizophrenia [6], borderline schizophrenia [2], and psychotic character [7] placed the phenomenon within the psychoses because of a presumed potential for psychotic decompensation; the syndrome remained poorly delineated, without a stable or widely shared definition.

The nosological history of the construct could be traced through the historical framework proposed by Gunderson, who outlines multiple phases in the evolution of the borderline concept, reflecting shifts in theory, research focus, and clinical attitudes, and organizes them into six periods [8]. In the first period, before 1970, borderline was used to describe patients viewed as being between neurosis and schizophrenia, with Kernberg’s model gradually influencing psychiatric discourse, sometimes dismissed within psychiatry as a psychoanalytic colloquialism [9]. A key advance in understanding borderline pathology nevertheless emerged precisely from Kernberg’s concept of Borderline Personality Organisation (BPO) [10], characterized by identity diffusion, primitive defenses centered on splitting (distinguishing it from neurotic organization), and preserved reality testing (distinguishing it from psychotic organization) [11]. Splitting is treated as a central clinical marker and a putative driver of cognitive, emotional, and behavioural manifestations: contradictory representations of self and object, emotional lability and impulsivity, and chaotic interpersonal relationships marked by primitive ambivalence and instability [12]. Grinker classified patients along a continuum from neurotic to psychotic and empirically identified four core elements of the borderline syndrome: anger, disturbed relationships, identity problems, and depressive loneliness [13]. In the second period (1970s–1980s), the focus shifted from personality organization toward a syndrome perspective, while Akiskal observed that “borderline is an adjective in search of a noun” [14]. During that period, BPD entered DSM-III and ceased to be conceptualized as a variant of schizophrenia, although speculations about atypical depression began to appear [14]. The third period (1980–1990) is marked by Vaillant’s remark that “it is wise never to call a patient borderline” [15] reflecting the accumulation of knowledge about countertransference difficulties in treating this group, while research simultaneously supported the consistency of the syndrome, its distinction from schizophrenia and depression, and a high rate of abuse histories (around 70%). In the fourth period (1990s), neurobiological models of affective dysregulation emerged, and the differential diagnosis broadened to include the bipolar II spectrum, raising the question of whether a patient could be considered borderline if they responded to medication, while the development of specialized psychotherapies partly supported the construct’s validity. In the fifth period (after 2000), BPD was framed as a “heritable brain disease with a good prognosis” [16]. The sixth period (from 2010 onward) can be summarized by the idea that “borderline patients have long been to psychiatry what psychiatry has been to medicine,” highlighting their neglected public health significance [8].

From 2010 onward, concerted efforts began toward the abolition of categorical models and the dimensionalization of PDs. These efforts were built upon earlier foundational work, including factor-analytic studies and critical reviews of categorical classification [17]. This period was marked by the acceptance of the Alternative Model for Personality Disorders (AMPD) as a proposal for further research in DSM-5 Section III, in which BPD was retained as one of six personality disorder categories alongside a dimensional trait framework, and by the work of the WHO ICD-11 Working Group on the Classification of Personality Disorders, whose first formal proposal for reclassification was presented by Tyrer et al. [18]. This process culminated in 2022 with the adoption of a fully dimensional classification of personality disorders in ICD-11, when all categorical diagnoses were abolished. However, a Borderline Pattern qualifier was preserved [19]. This period was also characterized by a convergence between personality and PD research, aligning dimensional models of personality pathology with the Five-Factor Model [20]. Following ICD-11 adoption, some Working Group members have argued that the Borderline Pattern qualifier is redundant, fully captured by severity and the trait domains of Negative Affectivity, Disinhibition, and Dissociality [21,22,23]. Authors opposing abandonment of the construct argue that doing so would constitute an unacceptable rupture with what is likely the most extensive documented clinical and research literature in the PD field [1,24,25,26,27].

The present review has three aims: first, to trace the nosological evolution of the borderline construct from early psychoanalytic descriptions through its operationalization in DSM-III and subsequent dimensional reforms in DSM-5 AMPD and ICD-11; second, to critically examine the principal arguments advanced for excluding borderline pathology from diagnostic classification systems and the evidence that complicates a straightforward conclusion of redundancy; and third, to propose a synthesis in which borderline pathology is reconceptualized as a clinically meaningful dimension of structural vulnerability and severity, a dimension that indexes the general factor of personality dysfunction, integrating the concept of the borderline level of personality functioning with the Level of Personality Functioning (Criterion A) of the DSM-5 AMPD and the ICD-11 severity continuum.

This narrative review draws on literature identified through searches of PubMed, PsycINFO, and Google Scholar, using terms including “borderline pathology,” “borderline personality organization,” “borderline personality disorder,” “borderline pattern descriptor,” “level of personality functioning,” “ICD-11 classification,” “severity,” and “general factor of personality disorder.” The time frame spans from foundational theoretical contributions (1938 onward) to contemporary publications (through early 2025), with emphasis on the last 15 years. As a narrative review, the literature selection was guided by conceptual relevance and the authors’ expertise rather than by systematic inclusion and exclusion criteria. Priority was given to foundational theoretical works on borderline personality organization, borderline level of functioning and dimensional models, WHO Working Group for ICD-11 Personality Disorders Classification publications, major longitudinal studies of BPD course and outcome, and recent empirical contributions to the dimensional classification debate. The review integrates historical, conceptual, and empirical perspectives, with the primary emphasis being conceptual, examining how the borderline construct has been understood across theoretical frameworks and what its place should be in contemporary dimensional systems.

## 2. Validity and Clinical Utility of the Borderline Construct

A research group associated with Peter Tyrer is among the most prominent advocates for excluding BPD from diagnostic classification systems, claiming that it lacks scientific validity. In ICD-11, the retention of a borderline pattern descriptor, notably not a formal diagnosis, is described by this group as a result of political compromise [19]. These authors cite three broad reasons for excluding BPD, and the term itself, from classification [22].

First, PDs are linked to maladaptive traits that are relatively stable over time, whereas the features used to describe BPD are not tied to a specific personality trait but rather to fluctuating symptoms and behaviours. As Mulder and Tyrer put it, BPD criteria are “not longstanding personality dispositions, but oscillating symptoms and behaviour.” A constant and undisputed diagnostic aspect of true personality disturbance is the presence of traits that are generally stable over time and, when disturbance becomes disorder, maladaptive. The widely fluctuating features of emotional instability do not belong in this paradigm [22].

Second, factor-analytic studies have not isolated a distinct borderline factor. The evidence suggests that BPD symptoms form a coherent syndrome when examined in isolation, but “disappear” into a general factor when criteria from other PDs are included. Sharp and colleagues identified a strong latent factor underlying the nine DSM BPD criteria. However, when symptoms of other PDs were incorporated into a model that also included a general factor, BPD items loaded entirely onto the general factor, and no specific borderline factor remained [28].

Third, beyond issues of diagnostic validity, these authors argue that the diagnosis lacks clinical utility: it is overly broad, applied non-selectively, and thus contributes to confusion and stigma, particularly among health professionals. They further question its usefulness on the grounds that it provides little guidance regarding specific treatment. In this view, specialized psychotherapies for BPD are better understood as transdiagnostic approaches, and no psychopharmacological treatment has demonstrated consistent disorder-specific efficacy. Indeed, the authors contend that the only accurate aspect of the diagnosis is its name, as it inadvertently points to the construct’s nonspecificity [22]. This raises a legitimate question: how should one conceptually organize the large body of clinical and research findings that continue to be recognized in everyday practice as “borderline,” findings that do not simply vanish when the diagnostic entity is removed from classification?

Having summarized the main arguments for excluding the borderline construct, we now turn to evidence that may complicate the straightforward conclusion that borderline pathology is merely redundant with severity and trait domains.

## 3. Level of Personality Functioning

One of the authors of this review (D.L.T.), a member of the WHO Working Group for ICD-11 Personality Disorders Classification, had previously developed, together with Divac-Jovanović and Svrakic, a dimensional model proposing that one core deficit in personality, broadly resembling Kernberg’s borderline personality organization, represents a common dimension extending across PD categories, whereas discrete PDs are categorical maladaptive behavioral types [29,30]. Their data suggested that discrete PDs share the borderline dimension, i.e., that borderline phenomena and features characterize the vast majority of subjects with PDs [29]. This approach was further elaborated in the proposal that PDs are disorders of adaptation rather than personality per se, since extreme personality traits are not ipso facto dysfunctional [31]. These foundational contributions, spanning three decades of clinical and empirical work [11,12,29,30,31,32,33,34,35], anticipated key features of the dimensional models now adopted in both DSM-5 AMPD and ICD-11.

Within this model, BPD is not treated as a discrete entity but as a level on a continuum between normal and neurotic functioning on one side and psychotic functioning on the other. The concept of continuum implies the absence of rigid boundaries, and functioning is viewed as dynamic rather than fixed, allowing for regressions and progressions across levels. Levels of functioning are described as relatively stable systems whose modulation depends on internal and external factors [12,33]. Borderline phenomena are framed as nonspecific markers of severity and dysfunction across PDs. Individuals with PD predominantly function at a borderline level of personality functioning while differing in trait-based types of adaptation (corresponding to trait-based descriptions) [29,31].

Within this framework, the concept of pseudo-borderline syndrome was introduced to describe a clinical presentation in which individuals whose baseline personality functioning is neurotic, characterized by integrated identity, mature defense organization, and intact reality testing, transiently exhibit borderline features (affective instability, diffuse identity, impulsive behavior, transient paranoid ideation) during the course of an affective disorder, most commonly major depression or dysthymia. Crucially, these borderline symptoms remit with successful treatment of the affective episode, revealing that the borderline presentation was state-dependent rather than reflecting a structural personality deficit [34,35]. The concept was developed on the basis of clinical observations that dysthymic disorder significantly distorts personality assessment, producing apparent borderline features that resolve when the affective condition is treated [35]. The clinical significance of this distinction extends beyond nosology: when affective and personality pathology coexist, as they frequently do, determining which is primary may be impossible on cross-sectional assessment alone, yet simultaneous improvement at both levels is needed if any progress is to be made [36]. The pseudo-borderline concept directly illustrates why the level of personality functioning must be understood as a dynamic dimension: the same surface symptoms may arise from fundamentally different structural substrates, with correspondingly different treatment implications and prognoses.

Regression proneness is defined here as a stable structural characteristic of borderline personality organization, reflecting the individual’s enduring vulnerability to transient shifts toward more primitive modes of functioning—primary-process thinking, activation of splitting and projective identification, disturbances in reality testing—under conditions of internal or external stress. Following Kernberg [10,37,38], what remains stable is the structural capacity for regression; what varies is only the occasion and intensity of its activation. Triggers for regression include interpersonal disruptions (perceived or actual abandonment, attachment threats), unstructured situations, therapeutic relationship ruptures, and affective episodes. This dynamic characteristic is one of the most clinically consequential features of borderline pathology—and a major source of treatment difficulty—yet it is omitted from the nine DSM-5 BPD criteria and from the ICD-11 borderline pattern specifier, and is even less likely to be captured by trait domain descriptors alone. Regression proneness has been empirically demonstrated through projective assessment: Rorschach Inkblot Test and Thematic Apperception Test (TAT) studies have consistently shown that BPD patients exhibit primary-process thinking with activation of primitive defenses and disturbed object representations when assessed with unstructured stimuli [1,39,40].

The two-step diagnostic logic described in this model, first assessing the level of personality functioning, then specifying the type of trait-based adaptation, aligns closely with contemporary dimensional models. In the AMPD, assessment begins with Criterion A, the Level of Personality Functioning (LPF), before proceeding to Criterion B, pathological trait domains. In the ICD-11, clinical decision-making similarly starts with determining whether a PD is present and, crucially, rating its severity, with trait domain qualifiers added as a secondary step. Both systems rate severity across graded levels that parallel the levels of personality organization described by Kernberg: moderate, severe, and extreme impairment in AMPD, and mild, moderate, and severe PD in ICD-11, correspond to high, middle, and low borderline personality organization, respectively [41,42]. This congruence is now supported by empirical convergence between measures of personality organization and both classification systems [43,44,45]. In each case, the level of personality functioning is primary for prognosis and treatment planning, while trait-based description is secondary, an approach that Kernberg’s concept of BPO and the concept of the borderline level of functioning had articulated decades earlier [29,30,41].

This convergence is consistent with the finding that borderline symptoms consistently load on the general PD factor in bifactor analyses. Sharp has argued that borderline criteria capture precisely what LPF is designed to measure, that is, self and interpersonal functioning, which is why they represent general personality pathology rather than forming a distinct specific factor [28]. Conceptually, the LPF domains of self-functioning (identity and self-direction) and interpersonal functioning (empathy and intimacy) correspond to the self and object representations that psychodynamic models place at the core of personality organization. This suggests that what psychodynamic tradition describes as borderline and what dimensional models measure as impaired personality functioning is the same underlying dysfunction, approached from different theoretical perspectives [46].

## 4. Discussion

### 4.1. Fluctuating Symptoms or Enduring Structural Vulnerability?

The stance toward borderline personality pathology varied not only between authors who supported or opposed its removal from diagnostic classifications, but also shifted within individual authors over time. Tyrer’s trajectory illustrates this evolution. Initially, he argued that BPD and schizotypal PD were unique among PDs, separated from more continuously distributed conditions by a “zone of rarity” a discontinuity in the PD spectrum that warranted their recognition as distinct clinical entities. Subsequently, he proposed reconceptualizing the condition as one of persistently unstable mood, coining the term “fluxithymia,” and recommended its reclassification within the affective disorders spectrum rather than outright elimination [47]. Finally, he called for abandoning the construct entirely as “spurious” and “unsupported by science” [22].

The three principal arguments advanced for excluding the borderline construct, that its features represent fluctuating symptoms rather than stable traits, that a distinct borderline factor does not emerge in factor analytic studies, and that the diagnosis lacks clinical utility, can each be examined on their own grounds.

The argument that borderline features are fluctuating symptoms rather than stable personality dispositions conflates two distinct levels of analysis. What fluctuates is the symptomatic manifestation, affective storms, impulsive acts, and transient psychotic episodes, but what remains stable is the underlying structural vulnerability that makes such fluctuations possible. The capacity for regression to a given level of dysfunction is itself a stable structural characteristic; what varies is only the occasion of its activation. This dynamic, in which an enduring vulnerability manifests episodically, is precisely what static trait domain profiles cannot represent. As outlined above, regression proneness is omitted from the nine DSM-5 BPD criteria, the ICD-11 borderline pattern specifier, and trait domain descriptors alike, yet it is one of the most clinically consequential features of borderline pathology and a major source of treatment difficulty [1,39,40]. Its triggers, ranging from interpersonal disruptions and attachment threats [48,49] through unstructured situations, therapeutic ruptures, and affective episodes, activate the same primitive modes of functioning documented by projective assessment. The pseudo-borderline syndrome [34,35] further illustrates that identical surface symptoms may arise from fundamentally different structural substrates, with correspondingly different treatment implications.

This dissociation between surface-level symptom change and enduring structural deficit has been empirically documented in major longitudinal studies. In the McLean Study of Adult Development, Zanarini and colleagues classified BPD symptoms into acute manifestations (rapidly remitting, such as self-mutilation and quasi-psychotic episodes) and temperamental features (persistent, such as chronic emptiness and abandonment concerns); while 93% of patients achieved symptomatic remission over 10 years, only 50% attained concurrent functional recovery [50,51]. Data from the Collaborative Longitudinal Personality Disorders Study yielded comparable findings: approximately 85% symptomatic remission but only 20% functional remission at 10 years [52]. Skodol and colleagues further demonstrated that functional impairment in BPD remained stable over two years despite some diagnostic improvement [53]. McGlashan et al. proposed a hybrid model in which personality disorders consist of stable traits and intermittently expressed symptomatic behaviors, a formulation that captures the clinical reality of enduring vulnerability with episodic symptomatic expression [54]. These convergent findings across independent longitudinal cohorts support the distinction drawn in the present review between fluctuating symptomatic manifestations and the stable structural vulnerability that constitutes the borderline level of personality functioning.

### 4.2. The Borderline Factor

The second argument, that a distinct borderline factor fails to emerge in factor analytic studies, is largely accurate but does not carry the implications its proponents assume. The absence of a specific borderline factor does not mean that borderline symptoms vanish from factorial solutions; rather, it reveals something fundamental about the nature of borderline pathology itself. Moreover, this claim is contradicted by the authors’ earlier findings. Although Mulder and Tyrer (2016) assert that such a factor never appears, a “borderline factor” did in fact emerge in a validation study of the five proposed ICD-11 trait domains conducted by the same group [55]. This factor predominantly comprised items from borderline, narcissistic, and histrionic PDs, aggregating a broad set of symptoms that merged dissociality and disinhibition while also correlating with negative affectivity and an antisocial factor. The authors themselves interpreted this factor as reflecting the heterogeneity of BPD and speculated that it might represent a general PD factor, as it included a wide spectrum of pathological traits and dysfunctional behavioural patterns across multiple PD types rather than being uniquely specific to BPD [55].

More broadly, the factor analytic literature reveals a consistent three-factor structure of PD pathology: an externalizing factor, an internalizing factor, and a detachment factor. Of these, the externalizing dimension is the most robust and clearly delineated. Borderline symptoms do not disappear in these solutions but recur in varying configurations, loading most frequently on the externalizing dimension alongside histrionic, narcissistic, antisocial, and paranoid symptoms [56,57,58]. Beyond the externalizing factor, borderline symptoms also load on an internalizing factor, where they co-occur with avoidant and dependent traits. In our own study revalidating the ICD-11 proposal using exploratory factor analysis, borderline symptoms did not disperse across other factors but instead formed a coherent factor together with avoidant and dependent features, which we termed a borderline internalizing factor [59]. When modelled within the broader structure of common mental disorders, BPD consistently loads on both the distress subfactor of the internalizing dimension and the externalizing dimension, a dual placement now codified in the Hierarchical Taxonomy of Psychopathology (HiTOP) model. As the HiTOP consortium has noted, BPD is “best considered an indicator of both internalizing and, to a lesser degree, the general externalizing superspectrum, likely with different components of the disorder being related to these two spectra.” [60].

Evidence from bifactor modeling clarifies why borderline features do not form a separate factor yet remain omnipresent across factorial solutions. As noted above, Sharp and colleagues [28] showed that borderline criteria load almost entirely on a general PD factor once other PD criteria are included. A large bifactor study by Jahng and colleagues, based on the U.S. National Epidemiologic Survey on Alcohol and Related Conditions (NESARC), further supports this view [61]. They identified a strong general factor spanning ten PD diagnoses that accounted for a substantial proportion of the comorbidity between PD and substance use disorders. After partialling out this general factor, a residual factor linked to Cluster B PDs remained, plausibly reflecting impulsivity or disinhibition, which showed an independent positive association with all substance dependence diagnoses across both sexes and age groups. With the residual factor controlled, the PDs loading most strongly on the general factor were avoidant, dependent, paranoid, and schizoid, conditions characterized by marked interpersonal dysfunction [61]. Borderline pathology thus operates simultaneously at two levels: it saturates the general factor that indexes overall severity of personality dysfunction, and its impulsive action components contribute to a specific externalizing residual. Similar results emerged from our study of a clinical PD sample, in which canonical analysis of covariance revealed a broad general PD factor predominantly driven by borderline, avoidant, dependent, detached, and anankastic traits and linked to high neuroticism and low conscientiousness, and a separate externalizing factor linking narcissistic, histrionic, and antisocial traits to high extraversion, high openness, and low agreeableness [62].

This cross-spectral placement receives independent support from research on higher-order personality metatraits. Building on Digman’s observation that the Big Five factors cluster into two higher order metatraits, Alpha (stability; N−, A+, C+) and Beta (plasticity; E+, O+), Strus and colleagues developed the Circumplex of Personality Metatraits (CPM), an octant model that maps both normal personality and personality pathology onto a common circular dimensional space [63,64]. In this model, internalizing PDs cluster around the maladaptive pole Gamma minus (N+, E−, O−, A−, C−), representing general dysfunction and disharmony, while externalizing PDs cluster around Delta minus (N+, E+, O+, A−, C−), representing disinhibition and sensation seeking. Borderline PD does not fall neatly into either cluster but occupies an intermediate position at the interface between the two, converging with the cross-spectral placement consistently observed in factor analytic and HiTOP studies [65,66].

### 4.3. Clinical Utility

The third argument concerns clinical utility: the borderline pattern is said to be applied non selectively, to offer limited clinically useful information, to generate confusion, and to be a source of stigma, and should accordingly be abandoned [21,22,23]. Given that ICD-11 includes a severity continuum and clearly defined trait domains, these authors argue that the borderline pattern adds no additional diagnostic information and that severity plus domain configuration can replace the descriptor entirely.

However, the empirical findings invoked in support of redundancy can equally be read as supporting a different conclusion. In a study published shortly before ICD-11 adoption, the same group examined overlap between BPD symptoms and total PD symptoms and found a strong association between hallmark borderline features, unstable identity and fear of abandonment, and overall PD severity [21]. The more severe the PD, the more likely borderline symptoms are to manifest. Rather than demonstrating that the borderline pattern is dispensable, this finding is consistent with the interpretation that it functions as a clinically recognizable marker of moderate to severe PD, precisely the role proposed by the concept of the borderline level of personality functioning [29] and by Sharp’s argument that borderline symptoms capture the general PD factor because they index impairments in self and interpersonal functioning [28,67,68].

The claim that the borderline pattern is fully captured by severity plus three trait domains rests on a compositional assumption: if a construct can be decomposed into its elements, the elements render the construct unnecessary. Several lines of evidence challenge this assumption. First, the decomposition is incomplete. Gutiérrez et al. found that ICD-11 trait domains accounted for only 65% of the variance in validated BPD measures, whereas the Borderline Pattern Scale explained 74%, a gap suggesting that domain-level descriptors do not fully capture what the borderline pattern indexes [69]. Second, the same configuration of domains can describe clinically distinct presentations: Negative Affectivity combined with Disinhibition and Dissociality may characterize a patient with identity diffusion, affective instability, and chronic self-harm, but equally one with substance dependence, anger dyscontrol, and intermittent antisocial behavior, presentations that differ in risk profile, treatment needs, and prognosis [25]. Third, borderline symptoms show incremental validity over trait dimensions in predicting functional outcomes: Morey et al. demonstrated that BPD features and Five Factor Model traits each predicted prospective functioning beyond what the other captured, with impulse action features such as suicidality and substance misuse showing relative independence from neuroticism. Descriptive overlap does not entail predictive redundancy [70].

Perhaps the most compelling practical argument for retaining the borderline construct lies in the treatment evidence. Borderline personality disorder is one of the few areas in psychiatry where disorder-specific psychotherapies have demonstrated efficacy in randomized controlled trials. Dialectical Behavior Therapy (DBT) [71], Mentalization-Based Treatment (MBT) [72], Transference-Focused Psychotherapy (TFP) [73], and Schema Therapy (ST) [74] have all shown significant reductions in self-harm, suicidal behavior, and core borderline symptoms. These treatments were developed specifically for borderline pathology; thus, abandoning the construct would risk severing the link between this evidence base and the clinical populations it was designed to serve. It may be argued that the therapeutic mechanisms of these treatments are transdiagnostic, applicable to eating disorders, self-harm, and other conditions beyond BPD. However, if borderline pathology indexes the general factor of personality dysfunction, as the present review argues, then the transdiagnostic reach of these interventions is precisely what would be expected: treatments targeting core personality dysfunction will inevitably benefit conditions that share that common substrate. The fact that they work across domains does not make the borderline construct redundant; it confirms its role as a central organizing principle. Of particular relevance, Kernberg’s model of expressive psychotherapy, now primarily known as Transference-Focused Psychotherapy, directly addresses the chaotic internal world of split self and object representations through here-and-now transference interpretation, while Kernberg also recommends supportive psychotherapy, applicable by trained psychotherapists, for patients with more severe borderline pathology characterized by fragile self-esteem, rudimentary superego integration, and preambivalent object relations [37,38]. Several of these interventions, particularly MBT and TFP, directly target impairments in self and interpersonal functioning (i.e., the Level of Personality Functioning), further supporting the convergence between the borderline construct and the severity dimension of personality dysfunction [1].

## 5. Conclusions

This review has argued that borderline pathology, far from being a spurious category to be ’buried,’ represents a clinically meaningful dimension that indexes the general factor of personality dysfunction—a dimension now formally recognized as Level of Personality Functioning in AMPD and as the severity continuum in ICD-11. The convergence between the concept of the borderline level of functioning and contemporary dimensional frameworks suggests that what has historically been called ’borderline’ is not a discrete disorder but a marker of severity and structural vulnerability, capturing impairments in identity, interpersonal functioning, and affect regulation that cut across PD types. That this construct ’dissolves’ into a general factor in structural analyses does not diminish its clinical utility. On the contrary, it reveals its nature as a reliable indicator of core personality dysfunction. Retaining borderline pathology as a recognizable clinical pattern within dimensional systems preserves continuity with an extensive treatment and research literature, including disorder-specific psychotherapies with demonstrated efficacy in reducing self-harm and suicidal behavior, provides clinicians with a configuration that specifies what severity looks like in practice, and maintains the dynamic perspective in which the capacity for regression is itself a stable structural characteristic, even when its manifestations are episodic.

### Limitations and Future Directions

As a narrative review, literature selection was guided by conceptual relevance and the authors’ theoretical perspective rather than systematic criteria; the analysis may not capture all relevant studies and may reflect interpretive emphasis inherent to psychodynamic and dimensional approaches. Research priorities include longitudinal testing of whether borderline features prospectively predict the trajectory of overall personality dysfunction, investigation of whether borderline pathology retains incremental predictive validity for treatment response, self-harm, and functional recovery beyond what severity ratings alone can capture, and examination of the relationship between the general factor of personality disorder and borderline pathology across diverse samples including adolescents where early identification may alter developmental course. The most pressing question is translational: dimensional systems now require clinicians to rate severity levels and trait domains, yet real-world data on how these ratings perform in routine practice remain scarce. It is still unknown whether severity and domain assessments can be reliably implemented across diverse clinical settings, including those with limited resources. In this context, the borderline pattern, with its long-established clinical recognizability, may serve as a practical anchor for severity assessment, offering clinicians a familiar configuration that specifies what moderate-to-severe personality dysfunction looks like in practice.

## Data Availability

No new data were created or analyzed in this study. Data sharing is not applicable to this article.
